# Evaluation of Complex Toxicity of Canbon Nanotubes and Sodium Pentachlorophenol Based on Earthworm Coelomocytes Test

**DOI:** 10.1371/journal.pone.0170092

**Published:** 2017-01-26

**Authors:** Yang Yang, Yao Xiao, Mei Li, Funian Ji, Changwei Hu, Yibin Cui

**Affiliations:** 1 State Key Laboratory of Pollution Control and Resource Reuse, School of the Environment, Nanjing University, Nanjing, China; 2 Shandong Provincial Key Laboratory of Water and Soil Conservation and Environmental Protection, Linyi University, Linyi, China; 3 Nanjing Institute of Environmental Sciences, Ministry of Environmental Protection, Nanjing, China; Chinese Research Academy of Environmental Sciences, CHINA

## Abstract

As a standard testing organism in soil ecosystems, the earthworm *Eisenia fetida* has been used widely in toxicity studies. However, tests at the individual level are time- and animal-consuming, with limited sensitivity. Earthworm coelomocytes are important for the assimilation and elimination of exogenous compounds and play a key role in the processes of phagocytosis and inflammation. In this study, we explored an optimal condition to culture coelomocytes of *E*. *fetida in vitro* and investigated the cytotoxicity of multiwalled carbon nanotubes (MWCNTs) and sodium pentachlorophenol (PCP-Na) using coelomocytes via evaluating lethal toxicity, oxidative stress, membrane damage, and DNA damage. The results showed that coelomocytes can be successfully cultured *in vitro* in primary under the RPMI-1640 medium with 2–4×10^4^ cells/well (1–2×10^5^ cells/mL) in 96-well plates at 25°C without CO_2_. Both MWCNTs and PCP-Na could cause oxidative damage and produce ROS, an evidence for lipid peroxidation with MDA generation and SOD and CAT activity inhibition at high stress. The two chemicals could separately damage the cell membrane structure, increasing permeability and inhibiting mitochondrial membrane potential (MMP). In addition, our results indicate that PCP-Na may be adsorbed onto MWCNTs and its toxicity on earthworm was accordingly alleviated, while a synergetic effect was revealed when PCP-Na and MWCNTs were added separately. In summary, coelomocyte toxicity in *in vitro* analysis is a sensitive method for detecting the adverse effects of carbon nanotubes combined with various pollutants.

## Introduction

Earthworms play a key role in nutrient mineralization, decomposition, and soil structure improvement [1]. They are considered as bioindicators of soil quality and health due to their sensitivity to various chemicals, such as nanomaterials, pesticides, and heavy metals [2, 3]. Therefore, standard methods have been established to measure the responses of individual earthworm species by determining mortality, behavior, pathological symptoms, body weight change, and reproductive activity [4–6]. However, large quantities earthworms were demanded for the observation of the end point of their death and it was necessary to consume much more chemicals in the experiment. Moreover, it takes a long time to prepare the artificial soil, e.g., the stable time and the exposure time of soil. Consequently, there has been attempted to develop more efficient methods to evaluate earthworm responses at a cellular level.

Coelomocytes, which are immunocompetent cells, play an essential role in the identification and elimination of xenobiotics [7], fighting microbial infections and healing wounds [8]. Due to their sensitivity to a wide range of pollutants, they have been widely used as one of the very sensitive biomarkers of environmental stress and are frequently used to assess genotoxicity [9, 10]. The most used approach has consisted of *in vivo* exposures and subsequent extrusion of coelomocytes from earthworms for the measurement of a variety of biomarkers [11, 12]. Compared with *in vivo* assays, *in vitro* exposures of coelomocyte primary cultures of *Eisenia fetida* have recently been implemented to determine toxicity profiles of different contaminants [13, 14]. However, most of these studies have either focused on cell recognition or cell culture condition, and systematic research in the field of *in vitro* toxicity is scarce. Although coelomocytes are increasingly being used for metal exposure, studies on nanoparticles and pesticide toxicity are limited [7, 9, 15].

Carbon nanotubes (CNTs), carbon allotropes with a cylindrical nanostructure, are currently attracting increased scientific interest because of their unique one-dimensional hollow structure and extraordinary mechanical, electrical, thermal, and optical characteristics [16]. However, applications using multiwalled carbon nanotubes (MWCNTs) are favored because of their lower production costs and because they do not have a critical carbon nanotube (CNT) diameter or band-gap, suggesting that the release of MWCNTs into the environment is highly possible [17, 18]. Carbon nanotubes have potential negative impacts on aquatic animals which take up nanomaterials through skin contact or orally through the gastrointestinal tract [18–20]. In addition, CNTs released into the environment will inevitably interact with other organic pollutants and influence their bioavailability as a carrier. There is still a lack of knowledge of the combined toxicity of CNTs and organic pollutants to terrestrial organisms. Thus, the measurement of the complex toxicity of MWCNTs and other pollutants has been an emerging issue for environmentalists.

Sodium pentachlorophenate (PCP-Na), as sodium salt of pentachlorophenol (PCP), poses a higher threat to waterways and estuaries via runoff from agricultural areas than industrial wastes, due to its high solubility [21, 22]. Although PCP-Na has been banned in China for a number of years now, its residues can still be found in the environment [2, 23].

To our knowledge, for the determination of coelomocytes in *in vitro* culture is still not being conducted systematically. Limited information of CNTs and PCP-Na toxicity on earthworm coelomocytes is available. This work explored the optimal conditions to culture coelomocytes of *E*. *fetida in vitro* and investigated the potential effect of PCP-Na, MWCNTs, and their complexes on coelomocytes under different exposure conditions in terms of cell survival rate, oxidative stress, membrane damage, and DNA damage, to provide valuable mechanistic information for ecological risk assessment for PCP-Na and MWCNTs.

## Materials and Methods

### Reagents

Sodium pentachlorophenate (PCP-Na, purity > 97%) was obtained from Sinopharm Chemical Reagent Co., Ltd. (Shanghai, China). 1000 mg/kg stock solutions were dissolved in distilled water to obtain the final concentrations of 0 (control), 10.67, 16, 24, 36, 54, 81, and 121.5 mg/L PCP-Na in the media. The MWCNTs, produced by chemical vaporization deposition (CVD), was purchased from Shenzhen Nanotech Port Co., Led. (Shenzhen, China).

### Cell extraction

Earthworms of the species *Eisenia fetida* were purchased from a farming factory in Jurong, Jiangsu Province, China. Healthy adult earthworms with a well- developed clitellum (60-days old, with a weight of 300–400 mg) were used for coelomocyte extraction after acclimation. The methods for cell extraction and toxicity measurement were based on the descriptions in Eyambe et al. (1991) with some modifications [24]. The earthworms were placed in enclosed petri dishes for 48 h on moist filter paper at 20 ± 1°C to purge their gut contents. Subsequently, individual earthworms were rinsed in cold extrusion medium (5% ethanol, 95% normal saline, 2.5 mg/mL EDTA, 10 mg/mL guaiacol glyceryl ether, pH 7.3) for 2 min. Coelomocytes were spontaneously secreted in the medium, the extrusion medium was then centrifuged at 4°C and 4,729 × g for 10 min) and the supernatant was removed. The coelomocytes were washed three times in phosphate-buffered saline solution (PBS). Survival rate was assayed by using the trypan blue exclusion method and a coelomocyte activity of at least 95% was cultured to test acute toxicity.

### Cell culture conditions

We selected four different media, low-glucose DMEM (Dulbecco minimum essential medium), high-glucose DMEM, RPMI-1640 (Roswell Park Memorial Institute), and DMEM-F12 to determine the culture conditions of coelomocytes. Two culture condition, 37°C with 5% CO_2_ and 25°C without CO_2_, were selected based on the imitation environment of common cell cultures *in vitro* and living earthworms, respectively. Cells were seeded into a 96-well plate and 200 μl of media were added to each well; inoculation density was 1 × 10^5^/ml, 2 × 10^5^/ml, and 5 × 10^5^/ml, with four replicates. Survival rate was measured using MTT 3-(4,5-dimethyl-2-thiazolyl)-2,5-diphenyl-2-H-tetrazolium bromide after 24, 48, and 72 h, respectively, for the optimum culture conditions.

### Toxicity under *in vitro* exposure

#### Acute toxicity of MWCNTs and PCP-Na

A 1.5-time concentration gradient of 0 (control), 10.67, 16, 24, 36, 54, 81, and 121.5 mg/L was prepared in the media to assess the LC_50_ of PCP-Na under the previously described optimum culture conditions. Two exposure methods were used in the combined acute toxicity examination: 1) “+”: MWCNTs and PCP-Na were separately added to the medium, and 2)“-”: MWCNTs coated with equal quantities of PCP-Na were added. Prior to the toxicity test, adsorption of PCP-Na onto MWCNTs was performed. For this, MWCNTs were dissolved in distilled water with final concentrations of 0 (control), 10.67, 16, 24, 36, 54, 81, and 121.5 mg/L PCP-Na and the mixture was covered and placed in a shaker (20°C, 250 rpm) for 24 h to ensure adsorption. The concentration of PCP-Na was measured and the amount adsorbed onto CNTs was calculated. The CNTs coated with equal quantities of PCP-Na were selected and air-dried for further use [23]. Subsequently, the stock solution containing 1,000 mg/L MWCNTs was placed in the ultrasonic cleaner and ultrasonicated with 80 percent power for 60 min to completely disperse MWCNTs. We diluted 50 mg/L MWCNTs for the combined toxicity study, for the toxicity of MWCNTs was slight based on data from the literature and on preliminary tests, with PCP-Na added simultaneously with the same concentration before. Cells were seeded into a 96-well plate with 200 μl media per well; inoculation density was 1 × 10^5^/ml, 2 × 10^5^/ml, and 5 × 10^5^/ml, with four replicates. Survival rate was detected by MTT after 24 h. We only considered data where the survival rate of the control group was at least 95%.

#### Oxidative damage to coelomocytes

The exposure treatments for oxidative damage to coelomocytes were conducted based on Table 1. Each treatment was performed in four replicates. Other steps were similar to those of the acute toxicity test.

**Table 1:** Dosages of MWCNTs and/or PCP-Na used for oxidative damage and membrane damage.

Exposure treatment	Abbreviation	MWCNTs (mg/kg)	PCP-Na (mg/kg)
Control	Control	-	-
MWCNTs_50_	M_50_	50	-
MWCNTs_200_	M_200_	200	-
PCP-Na_1/20_	P_1/20_	-	5.22 (1/20 LC_50_)
PCP-Na_1/10_	P_1/10_	-	10.44 (1/10 LC_50_)
PCP-Na_1/5_	P_1/5_	-	20.88 (1/5 LC_50_)
MWCNTs_50_ + PCP-Na_1/20_	M + P_1/20_	50	5.22 (1/20 LC_50_)^a^
MWCNTs_50_ + PCP-Na_1/5_	M + P_1/5_	50	20.88 (1/5 LC_50_)^a^
MWCNTs50—PCP-Na_1/20_	M—P_1/20_	50	5.22 (1/20 LC_50_)^b^
MWCNTs50—PCP-Na_1/5_	M—P_1/5_	50	20.88 (1/5 LC_50_)^b^

^a^ MWCNTs and PCP-Na added separately

^b^ MWCNTs coated with PCP-Na were added.

The cell suspension was re-suspended in a 96-well plate and then transferred to a 1.5 mL Eppendorf vial. The supernatant was removed after centrifugation at 1,500 rpm, 4°C for 10 min, and the obtained pellet was washed three times and re-suspended in 1 ml PBS. After ultrasonication (0.5 s: 1 s, work: interval) for 30 s in an ice bath, the cell suspension was again centrifuged at 3,000 rpm and 4°C for 10 min; the supernatant was transferred to a new tube for physiological and biochemical index analysis.

Superoxide dismutase (SOD), catalase (CAT) activities, and malonaldehyde (MDA) were measured using commercial kits (Jiancheng Co. Ltd., Nanjing, China). Reactive oxygen species (ROS) were determined using the ROS assay kit (Beyotime Institute of Biotechnology, China). Enzyme activities were standardized by protein content. Each experiment was performed in triplicate.

#### Membrane damage

The exposure method and exposure treatments both followed the protocols for evaluation of coelomocyte oxidative damage, including cell homogenate preparation. Lactate dehydrogenase (LDH) activity was measured using commercial kits (Jiancheng Co. Ltd., Nanjing, China) and determination of mitochondrial membrane potential (MMP) was carried out using a cell apoptosis detection kit (Rhodamine 123) (KeyGEN BioTECH, Nanjing, China).

#### DNA damage

The exposure method followed the protocol for determining coelomocyte acute toxicity. Higher concentrations of MWCNTs, 250 and 500 mg/kg l was choosen for single treatment as to the MWCNTs toxicity was relatively low, while a lower concentration of PCP-Na_1/50_ (2.09 mg/L) was chosen for DNA damage, with DNA being sensitive to various environmental contaminants. Each treatment was performed in four replicates. Coelomocytes were obtained according to the non-invasive extrusion method described by Eyambe et al. [24]. The level of DNA strand breaks was determined using the method described by Singh et al. [25]. After electrophoresis, slides were washed three times with 0.5 M Tris buffer (pH 7.5) and DNA was stained with ethidium bromide. Slides were examined with a fluorescent microscope (BX41, Olympus, Japan). A total of five groups were examined, with three slides per treatment, and at least 50 cells were analyzed for each slide. Photos were taken with a digital camera (C-5050ZOOM, Olympus, Japan) and images were analyzed by CASP software according to the method of Collins et al. [26].

### Statistics analyses

Statistical differences of biological parameters between each treatment, including the control, were evaluated using one-way analysis of variance (ANOVA), followed by a Dunnett *t* (2-sided) test. Results were expressed as means ± standard deviation (SD). All analyses were performed using SPSS 16.0 software and the probability level for statistical significance was *p* < 0.05.

The LC_50_ (median lethal concentration) values were calculated using the regression line obtained by plotting the concentration against the death percentage on a probit scale and the results were evaluated with probit analysis (SPSS 17.0), using the following equation [14]:

$$LC_{50}=LC_{100}\;\text{-}\;\left(ab+\ldots +ab\right)/n,$$

where LC_50_ is the concentration with a survival rate of 50% of the coelomocytes and LC_100_ is expressed as percentage values relative to the signal given by control coelomocytes (100%). The variables a, b, and n are the difference between two consecutive doses.

## Results

### Cell culture condition

**Fig 1:**
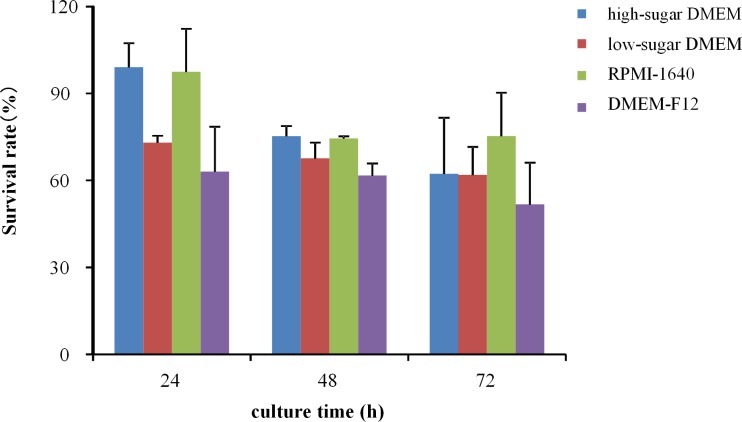
Coelomocytes survival rate in 72 h incubated under optimal culture condition

After coelomocyte extraction, coelomocyte activity was over 95% in terms of survival rate measurement of trypan blue exclusion. Survival rates of coelomocytes under different conditions are shown in S1 and S2 Figs. The results show that the coelomocytes can be suspension-cultured *in vitro*. Cell survival rate of coelomocytes was lower than 30% after cultivation of 24 h at 37°C with 5% CO_2_ (S1 Fig), cultivation at 25°C without CO_2_ was showed to a more suitable method. Compared with 48 h and 72 h observation of the optimum culture condition, optimum exposure time was 24 h. Cell survival rate was almost 100% in 24 h for high-glucose DMEM and RPMI-1640 medium, but after 72h exposure, cell survival rate of RPMI-1640 medium was still over 80%, suggesting that RPMI-1640 is more suitable for coelomocyte cultivation. Optimal culture conditions were RPMI-1640 medium with 2–4 × 10^4^ cells/well (1–2×10^5^ cells/mL) in a 96-well plate at 25°C, without CO_2_ (Fig 1).

### Acute toxicity

In regards to the acute toxicity test, LC_50_ of PCP-Na was 104.46 mg/L (Table 2). The toxicity test of PCP-Na combined with 50 mg/L CNTs was conducted when the MWCNTs and PCP-Na were added separately; LC_50_ of PCP-Na was 109.39 mg/L, and at the condition that PCP-Na was absorbed by CNTs before addition, LC_50_ of PCP-Na was 112.92 mg/L. The acute toxicity of PCP-Na was slightly reduced after combining with CNTs. Acute toxicity of CNTs was very low and we did not obtain an LC_50_ value, which is consistent with the acute toxicity test results on an individual level [22].

**Table 2:** Acute toxicity of PCP-Na to coelomocytes.

	PCP-Na	PCP-Na + CNTs	PCP-Na—CNTs
LC_50_(mg/L)	104.46	109.39	112.92
95% confidence interval	85.62–137.21	90.42–133.17	79.37–166.17

+): MWCNTs and PCP-Na added separately

-): MWCNTs coated with PCP-Na were added.

### Oxidative damage

**Fig 2:**
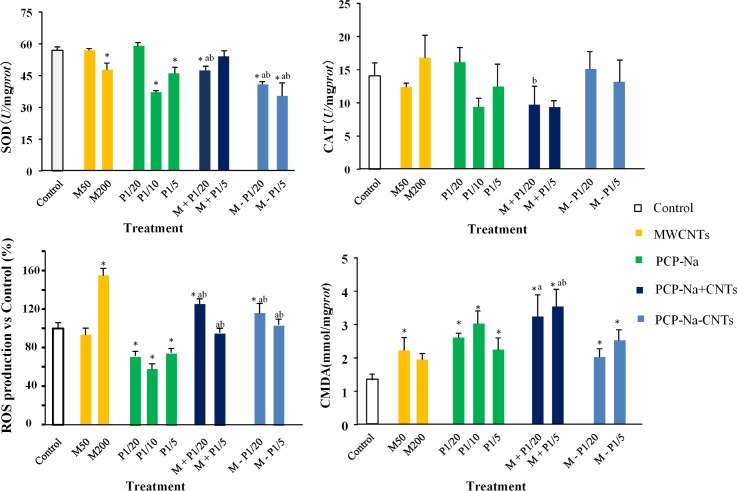
Activities of antioxidant enzymes of the earthworm coelomocytes after exposure to different treatments of MWCNTs (M), PCP-Na (P), and their complex (A) SOD, (B) CAT, (C) ROS, and (D) MDA. Data are expressed as the mean ± standard deviation (SD) of quadruplicate analyses. ^*^ means which has a significant difference from the control (CK), ^a^ means the complex group which had significant difference from M50, ^b^ means the complex group which had significant difference from corresponding P1/20 or P1/5 (*p* < 0.05).

The levels of SOD, CAT, ROS, and MDA in coelomocytes were measured after 24-h exposure to characterize the changes of antioxidant defenses induced by MWCNTs and PCP-Na (Fig 2). The results show that SOD activities significantly (*p* < 0.05) decreased in groups with PCP-Na combined with 50 mg/L CNTs compared to the control group (Fig 2A). Moreover, adsorption of MWCNTs and PCP-Na could inhibit SOD activity more effectively compared with separate addition. The activity of CAT has no significant (p > 0.05) difference between the control group and treatment group except for the separately co-added group M + P_1/20_ and M + P_1/5_ (Fig 2B). The ROS content decreased significantly (*p* < 0.05) after PCP-Na exposure, but increased significantly in 200 mg/L MWCNTs or M + P_1/20_, M—P_1/20_ (Fig 2C). The MDA level significantly increased after MWCNTs and PCP-Na exposure with higher adsorption values for separately added substances (Fig 2D).

### Membrane damage

**Fig 3:**
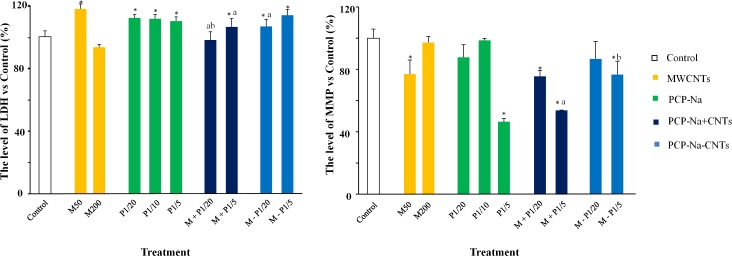
Activities of antioxidant enzymes of the earthworm coelomocytes after exposure to different treatments of MWCNTs (M), PCP-Na (P), and their complex (A) LDH, (B) MMP. Data are expressed as the mean ± standard deviation (SD) of quadruplicate analyses. ^*^ means which has a significant difference from the control (CK), ^a^ means the complex group which had significant difference from M50, ^b^ means the complex group which had significant difference from corresponding P1/20 or P1/5 (*p* < 0.05).

We measured LDH and MMP to assess the membrane damage caused by MWCNTs and PCP-Na (Fig 3). The results of LDH activity and MMT in coelomocytes are shown in Fig 3; LDH activity was significantly promoted after 24-h exposure to various concentrations of PCP-Na with or without MWCNTs, except for M + P_1/20_ (Fig 3A).

The results show that the MMP level significantly (*p* < 0.05) decreased in treatment groups of PCP-Na combined with 50 mg/L CNTs compared with the control (Fig 3B). Moreover, separate addition of MWCNTs and PCP-Na could inhibit MMP levels more efficiently after adsorption, indicating that mitochondria suffered damage under MWCNTs and PCP-Na exposure.

### DNA damage

In each treatment, earthworm coelomocyte DNA suffered evident damage compared with the control, indicating that the comet assay is sensitive enough to evaluate earthworm DNA damage caused by PCP-Na and CNTs (Table 3). When exposed to CNTs, there was a significant dose-response relationship between CNTs and DNA damage. In single PCP-Na groups, the higher concentration of PCP-Na had lower DNA damage. DNA damage showed more serious in higher PCP-Na exposure group (50 + 1/5 LC_50_) when MWCNTs and PCP-Na were added separately or MWCNTs coated with PCP Na added. MWCNTs coated with PCP-Na added, which is in accordance with other biochemical parameters, such as ROS and MDA.

**Table 3:** DNA damage of earthworm coelomocytes under PCP-Na and MWCNTs exposure.

Treatment(mg/kg)		Tail DNA%	Tail length	OTM
CK		8.87 ±0.67	29.22 ±7.46	4.24±0.33
	50	22.62 ± 1.31*	64.26 ± 5.75*	11.09 ± 1.67*
CNTs	250	24.91 ± 0.73*	79.25 ± 3.47*	12.97 ± 1.06*
	500	36.93 ± 3.23*	105.47 ± 14.43*	19.01 ± 1.85*
PCP-Na	1/50 LC_50_	33.43 ± 2.41*	84.67 ± 3.80*	19.10 ± 1.01*
	1/5 LC_50_	24.28 ± 1.56*	76.71 ± 5.04*	15.63 ± 1.02*
CNTs + PCP-Na	50+1/50 LC_50_	33.60 ± 1.42*^a^	99.53 ± 5.13*^a^^b^	20.56 ± 1.67*^a^
	50+1/5 LC_50_	39.07 ± 2.06*^a^^b^	108.72 ± 12.27*^a^^b^	27.16 ± 1.25*^a^^b^
CNTs—PCP-Na	50-1/50 LC_50_	36.87 ± 3.19*^a^	89.77 ± 10.48*^a^^b^	23.90 ± 4.24*^a^^b^
	50-1/5 LC_50_	29.91 ± 2.00*^a^^b^	82.47 ± 5.97*^a^	16.55 ± 1.61*^a^

Data represent mean ± standard deviation (SD) of quadruplicate analyses.

* indicates significant difference from the control (CK)

^a^ complex group with significant difference from M_50_,

^b^ complex group with significant difference from corresponding P_1/20_ or P_1/5_ (*p* < 0.05).

## Discussion

Cell survival rate of coelomocytes was lower than 30% after cultivation of 24 h at 37°C with 5% CO_2_. Cultivation at 25°C without CO_2_ was a more suitable method. A previous study has shown that the optimum temperature for earthworm growth was 20–30°C, with a pH range between 7 and 8 [27]. The presence of 5% CO_2_ might decrease the pH of the medium, resulting in unsuitable conditions for cell growth. Cell survival rate decreased when inoculation density exceeded 1 × 10^5^/ml, possibly due to increased metabolic requirements [14]. Cell survival rate was almost 100% in 24 h for high-glucose DMEM and RPMI-1640 medium, but after 72h exposure, cell survival rate of RPMI-1640 medium remained over 80%, suggesting that RPMI-1640 was more suitable for coelomocyte cultivation. In addition, our results indicate that 24 h incubation rendered the highest and the less variable signal in overtime culture. Incubation time is critical and differs substantially between earthworm species [28]. Accordingly, the cells were thus incubated for 24 h at room temperature (21°C) in darkness after *in vivo* exposure to nanoparticles [13].

Based on the 24-h exposure test for coelomocytes, the LC_50_ of PCP-Na was 104.46 mg/L. Acute toxicity of PCP-Na in the combined treatments with MWCNTs was lower than that of PCP-Na alone, while there was no significant difference between the group of PCP-Na alone and PCP-Na co-added with MWCNTs. This is not in agreement with previous results showing that the toxicity of PCP-Na for *E*. *fetida* was alleviated by the appearance of MWCNTs in the filter paper contact test and the artificial soil contact test [22, 29]; this was probably due to the fact that the acute toxicity response was not sensitive enough to evaluate earthworm damage caused by PCP-Na and CNTs in coelomocytes toxicity.

Oxidative stress is a common mechanism involved in the cytotoxicity of CNTs [30, 31]. Antioxidant defenses consist of different enzymes, including SOD, CAT, and GSH-Px. Based on the results of previous studies, the biochemical parameters SOD and CAT were chosen to evaluate the responses to various environmental contaminants [32]. In this study, SOD activity decreased significantly at M_200_, which showed that coelomocytes still suffered from oxidative stress, although the acute toxicity of MWCNTs was quite low. The PCP-Na also caused significant inhibition over the concentration of 10.44 mg/L (1/10 LC_50_), both MWCNTs and PCP-Na showed a significant dose-response relationship (*p* < 0.05) with SOD. However, in a similar study, Zhang et al. [33] discovered that SOD activity was significantly stimulated by the highest dose (2.5 mg/kg) of spirotetramat for the entire period of exposure. In our study, MWCNTs and PCP-Na showed more serious effects on coelomocytes when they were added after adsorption, which is in contrast to the other biochemical parameters. In a study by Wei et al. [34], SOD activity was elevated first as a direct response to of the increased amount of superoxide anion radicals. The underlying mechanism for this might be that the natural antioxidant defenses were not sufficient anymore [22]. Catalase has a sufficient capacity for decomposing excess H_2_O_2_ to protect cells from oxidative stress. Some isozymes, such as POD and GPX, perform the same function in scavenging H_2_O_2_ [35, 36]. In this study, CAT activity showed no significant change in any treatment group compared with the control, which might be related to the hydrogen peroxide transformation mechanism that POD played a significant role in deposing the excess H_2_O_2_ during this treatment.

In addition, we characterized the cell response to PCP-Na and MWCNTs by examining ROS production and lipid peroxidation. Previous studies have demonstrated that ROS can be generated in earthworms exposed to stress caused by environmental contaminants [22, 37]. Our study showed that high concentrations of MWCNTs (200 mg/kg) or low concentrations of PCP-Na combined with MWCNTs (50 mg/kg) induced ROS production. Overproduction of ROS can lead to oxidative damage to macromolecules such as nucleic acids, proteins, and lipids, eventually resulting in cell damage [38]. In the present study, the MDA content in the treatment groups was higher than in the control group throughout the exposure time, indicating that more products of peroxidized unsaturated fatty acids had accumulated in tissues. Separately added MWCNTs and PCP-Na induced more serious lipid peroxidation damage compared with MWCNTs coated with PCP-Na. In a previous study, cytotoxicity was enhanced by two to three times when the biphenyl compounds were combined with SWCNTs [39]. However, several studies have demonstrated that MWCNTs or other carbon materials could reduce the bioavailability of chemicals or inhibit their toxicity [40–42]. For our study, the underlying mechanism for this might be that MWCNTs are effective adsorbents of organic chemicals and could adsorb PCP-Na onto the surface. When MWCNTs and PCP-Na were added separately, small detached molecules promoted higher effects on cells, thereby revealing a synergetic effect inducing more serious lipid peroxidation damage. After adsorption, PCP-Na combined preferentially with MWCNTs instead of coelomocytes, reducing bioavailability of chemicals or inhibiting their toxicity compared with separately co-added.

In response to excessive ROS, endothelial functions were impaired and even apoptosis was triggered [43]. Lactate dehydrogenase (LDH) is an important metabolic dehydrogenase which catalyzes the interconversion of pyruvate to lactate in anaerobic glycolysis [44]. It is a cytoplasmic enzyme involved in anaerobic energy production and a sensitive index of the loss of cell membrane integrity in nanotoxicology studies [44]. Both PCP-Na and MWCNTs (50 mg/L) could damage the membrane structure of the cell, increasing permeability. The inhibition of LDH activity in the 200 mg/L MWCNTs treatment might be due to the binding of MWCNTs or its metabolite(s) with the enzyme molecules or by blocking enzyme synthesis [45]. Although PCP-Na and MWCNTs showed an antagonistic effect on LDH activity for the combined groups, while the phenomenon was not significant, it might be due to the oversaturated LDH released into the media, resulting in inhibition of LDH under co-exposure [46]. From this, it can be deduced that MWCNTs and PCP-Na revealed a synergetic effect, inducing more serious cytomembrane damage when separately added, based on LDH activity.

The mitochondrion is one of the most important compartments for ROS generation in cells; mitochondrial disorder is a key step during apoptosis [47]. A variety of studies have shown that apoptosis was often associated with loss of the mitochondrial inner potential, and the reduction of MMP was among the changes encountered during the early of apoptosis [44]. As shown in Fig 3, after incubation with high concentrations of PCP-Na, 50 mg/L MWCNTs, and combined compounds, the MMP showed a significant decrease, indicating that both of them can induce cell apoptosis through mitochondrial impairment. Dong et al. [48] found that PCP-induced apoptosis occurred in a dose-dependent manner with the mitochondrial membrane potential disruption and lipid peroxidation production (MDA) increase. In our study, MMP decreased most conspicuous with 20.88 mg/L PCP-Na (1/5 LC_50_), and the addition of MWCNTs reduced mitochondrial damage caused by PCP-Na, which was consistent with the acute toxicity result. This finding might be explained by toxic chemical kinetics. Based on our previous studies, MWCNTs show a rapid adsorption rate and promising removal efficiency for PCP-Na; PCP-Na uptake can be significantly (*p* < 0.05) decreased in the presence of MWCNTs, resulting in less PCP-Na accumulation in cells and thus reduced mitochondrial damage.

The comet assay has been widely used for nanotoxicology research on *E*. *fetida* and is a useful tool to identify genotoxic effects of chemicals on invertebrates [23, 32, 49]. Tail DNA% (tDNA), olive tail moment (OTM), and tail length increased significantly (*p* < 0.05) under PCP-Na and MWCNTs treatment compared with control groups, demonstrating that both of them caused DNA damage to coelomocytes (Table 3). In contrast, there was a significant dose-response relationship between MWCNTs and DNA damage, while higher concentrations of PCP-Na showed lower DNA damage after exposure. This is in agreement with the findings of Hu et al. [23], who reported that high dosages (14.2 mg/kg) of PCP-Na induced significantly lower (*p* < 0.01) values of the parameters compared with lower dosages (1.4 mg/kg). Martínez et al. [50] also found no differences in the extent of DNA fragmentation related to the concentration of the chemical, and DNA damage was barely affected by exposure time. Lower extent of DNA damage at 20.88 mg/L (1/5 LC_50_) may be due to the fact that most of the severely damaged DNA broke into small fragments and was lost during electrophoresis procedures of the comet assay, and only the slightly damaged DNA was analyzed in the higher dosage treatments. However, the mechanisms responsible for genotoxicity of PCP-Na to cells or animals are not clear. After 24-h exposure, the toxicity induced by MWCNTs and PCP-Na in combination was enhanced more or less compared with MWCNTs (50 mg/kg) and PCP-Na (2.09 and 20.88 mg/kg) individually, especially when added separately. The adsorption of MWCNTs to PCP-Na might lessen the toxicity of PCP-Na due to the PCP-Na absorbed by MWCNTs; the smaller amount of hydrogen peroxide produced can thus be eliminated by the cell´s normal antioxidant enzymes [22]. Overall, DNA damage in each treated group was significantly different compared with the control, indicating that the comet assay has sufficient sensitivity to evaluate earthworm DNA damage caused by PCP-Na and CNTs.

## Conclusions

Cell survival rate, oxidative stress, membrane damage, and DNA damage of coelomocytes exposed to PCP-Na, MWCNTs, and their complex *in vitro* were associated with exposure concentration and methods. Overall, both CNTs and PCP-Na can cause oxidative damage and produce ROS, resulting in lipid peroxidation with MDA generation and SOD and CAT activity inhibition at high stress, which is consistent with the individual results. In comparison, higher susceptibility was obtained in *in vivo* cytotoxicity tests in this study, compared to traditional endpoints. Moreover, CNTs and PCP-Na could damage the membrane structure of the cells, increasing permeability and inhibiting MMP. In addition, our results indicate that the toxicity of PCP-Na may be alleviated by the appearance of MWCNTs after adsorption, while PCP-Na and MWCNTs added separately had a synergetic effect based on the cytotoxicity toxicity study. In summary, coelomocyte toxicity *in vitro* analysis is a new sensitive method for detecting the adverse effects of various pollutants on earthworms, and the cytotoxicity indexes are positively correlated with the indexes for individual levels. Earthworm cytotoxicity tests are expected to replace individual level toxicity tests and can become a fast, efficient, and sensitive method to evaluate toxicity of pollutants on soil organisms.

## Supporting Information

S1 Fig Coelomocytes survival rate after 24 h incubated in 37°C. (TIF)

S2 Fig Coelomocytes survival rate after 24 h incubated in 25°C. (TIF)
