# Supplementary figures and images for: Evaluation of Complex Toxicity of Canbon Nanotubes and Sodium Pentachlorophenol Based on Earthworm Coelomocytes Test
Source: PLoS One. 2017 Jan 26;12(1):e0170092. doi: 10.1371/journal.pone.0170092 (PMC5268766; doi:10.1371/journal.pone.0170092)

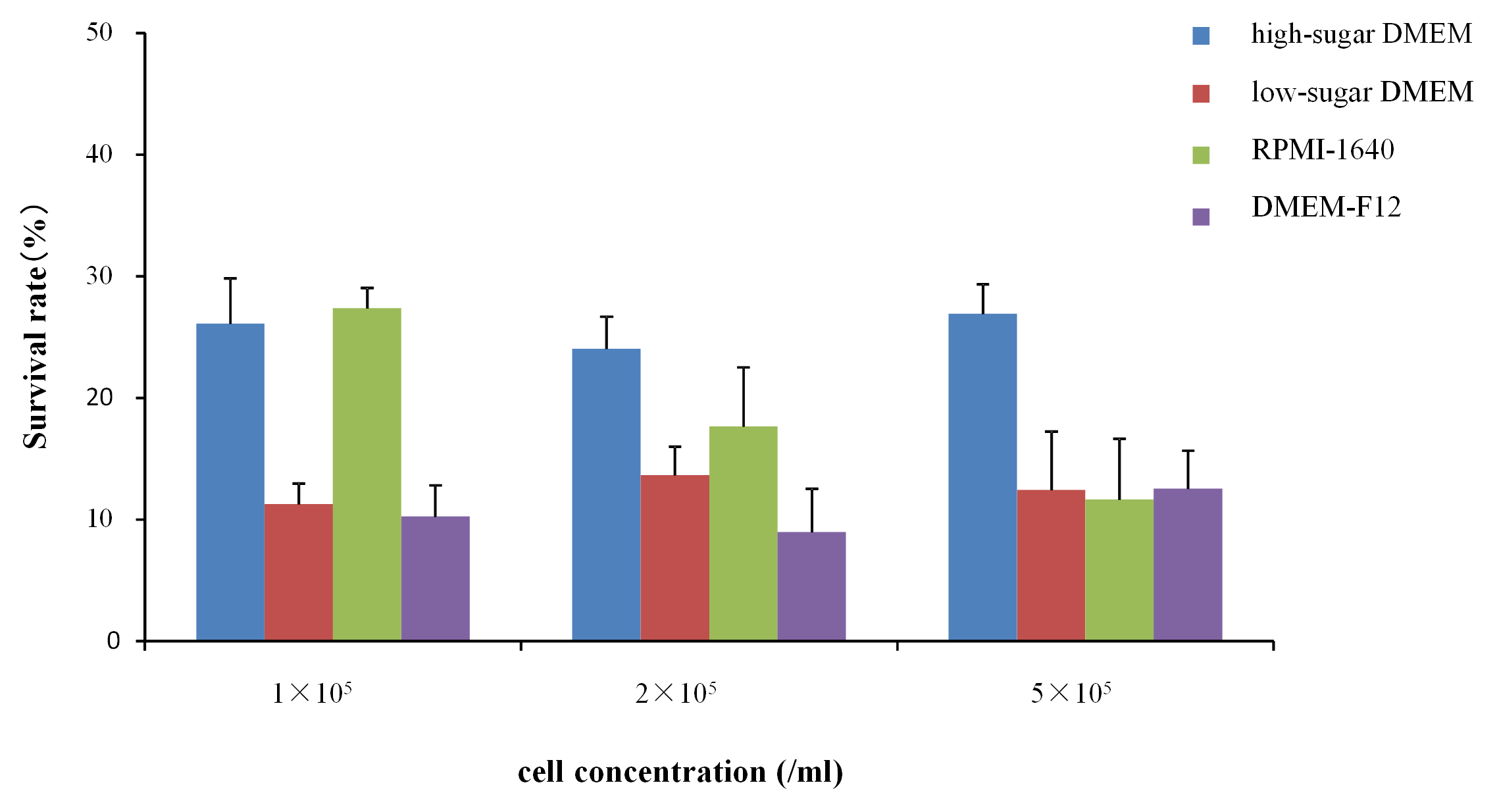

Supplement: S1 Fig — (TIF) [file pone.0170092.s001.tif]

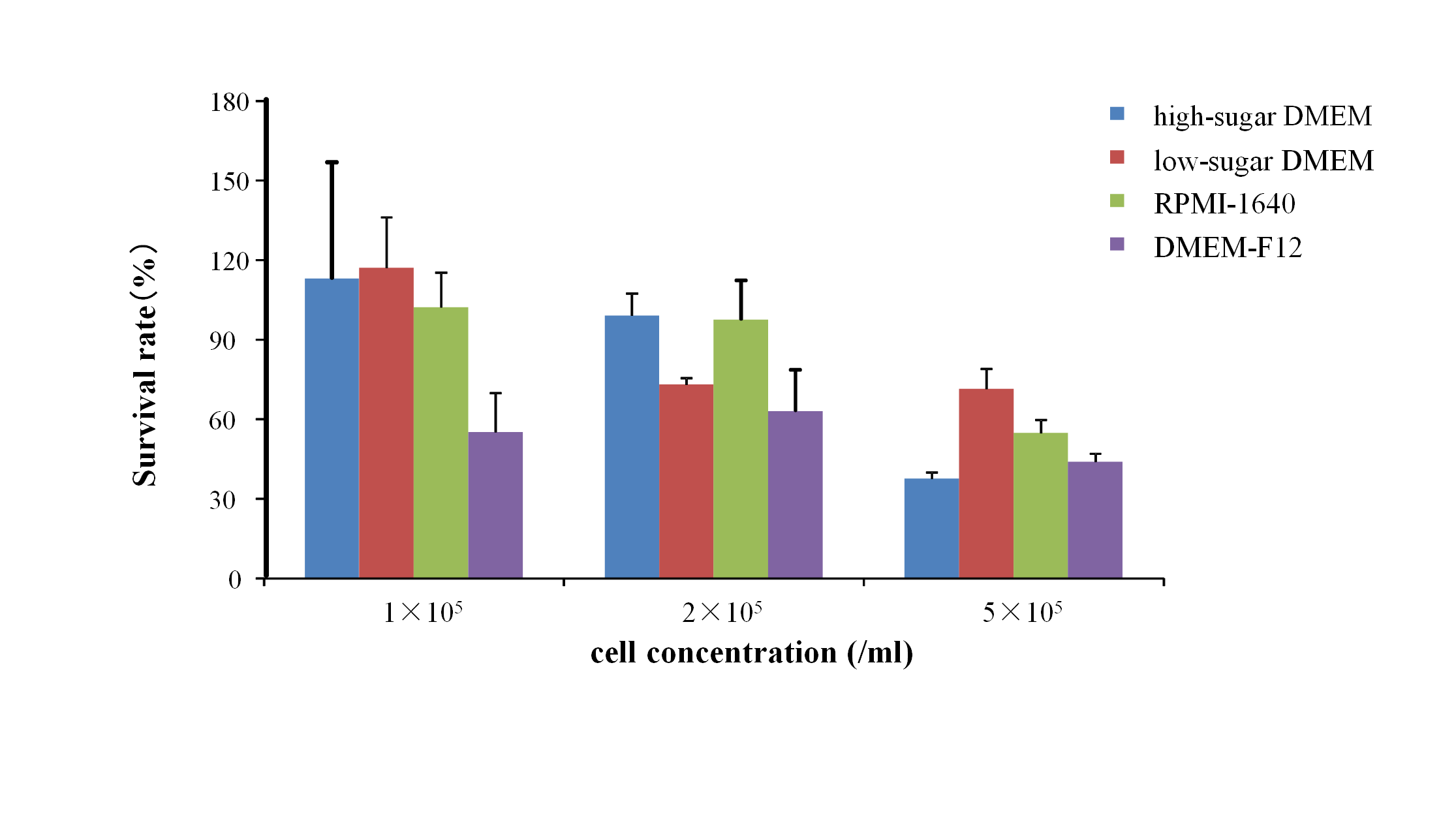

Supplement: S2 Fig — (TIF) [file pone.0170092.s002.tif]
